# Typhoid in Bangladesh: Challenges, efforts, and recommendations

**DOI:** 10.1016/j.amsu.2022.104261

**Published:** 2022-07-31

**Authors:** Yumna Salman, Hanzla Asim, Narmeen Hashmi, Zarmina Islam, Mohammad Yasir Essar, Md Ariful Haque

**Affiliations:** Faculty of Medicine, Dow Medical College, Dow University of Health Sciences, Pakistan; DHQ Teaching Hospital Dg khan, Pakistan; Faculty of Medicine, Dow Medical College, Dow University of Health Sciences, Pakistan; Kabul University of Medical Sciences, Kabul, Afghanistan; BTF Medical Institute, Satkhira, Khulna, Bangladesh; Department of Public Health, Atish Dipankar University of Science and Technology, Uttara, Dhaka, Bangladesh

## 1 Introduction

Typhoid fever, a highly contagious acute infection caused by the gram-negative bacterium, *Salmonella enterica* serotype Typhi (S.typhi) [1].It is transmitted through faecal-oral route or through ingestion of undercooked food and water [1] (notably unboiled tap water) [2].Other associated methods include shedding of S.typhi by either an infected, or asymptomatic individual who has not maintained good hand hygiene, hence would result in contamination of new food and water [3] causing spread of typhoid to healthy individuals.

S.Typhi commonly associated with prolonged fever ranging as high as 103–104 °F (39–40 °C) [4]. Other symptoms include headache, cough, extreme fatigue, constipation, loss of appetite and diarrhoea (the latter two occur in later stages of the infection) [5]. Symptoms typically arise 1 or 2 weeks after being infected [5] and if left untreated can result in intestinal and neuropsychiatric complications [6].

According to the World health organisation (WHO); 11–20 million typhoid cases are reported globally with mortality of 128,000–161,000 deaths per annum [7]. Bangladesh amongst many others is greatly impacted with incidence rates of 252 per 100,000 people affected yearly [8]. Children-especially immunocompromised [1], under five are remarkably more vulnerable as compared to older individuals [9]. Poor communities lacking clean food, water [1] and adequate sanitation are also prone [7]. Multiple climate factors like increased rainfall, river levels and temperature [10] has shown to increase typhoid distribution typically in Bangladesh that receives an average of 2,200 mm (mm) of rainfall per annum [11]. This causes a formidable strain on the water, sanitation, and hygiene infrastructures as problems like drain blockage and improper drainage of wastewater leads to contamination of shallow water sources; these are normally used in times of droughts and individuals living in rural areas or slums [12]. Moreover, escalation of typhoid cases is only exacerbated in densely populated countries like Bangladesh where individuals are exposed to a single contaminated source.

Furthermore, the surfacing of the pandemic has resulted in emergence of co-epidemics which has only further burdened the already strained healthcare system. The Global Burden of disease study estimated that typhoid has consumed the lives of more than 110,000 individuals globally with around 9 million cases reported yearly [13,14]. 7,100 positive S. typhi cases were noted during 2001–2014 [15] prior to the pandemic, in comparison to this, during the pandemic, in 2021,1,135 typhoid cases per 100,000 were recorded [13]. Additionally,1,953,103 COVID-19 cases were confirmed between January 3, 2020 to May 20, 2022 [16] in Bangladesh. Consequently, this highlights the economic burden placed on healthcare systems as much of the focus has shifted to distribution and management of the highly virulent coronavirus which was spreading like wildfire. Moreover, another difficulty which healthcare professionals are facing is differentiating between both diseases (COVID-19 and typhoid) as they present with similar nonspecific symptoms which include fever, fatigue, and body aches [17]. This makes it troublesome to identify the true underlying cause without proper diagnostic testing. This article aims to focus on the current situation of typhoid in Bangladesh and suggest constructive methods which can be implemented to avert its spread in Bangladesh.

## 2 Challenges and implications

Various challenges are being faced by Bangladesh leading to difficulties in limiting typhoid. These include the water problems, its availability and safety to use, the unhygienic street foods and poor healthcare infrastructure. Another important challenge being faced on the part of the organism is its resistance to antibiotics.

According to the World Health Organisation, water is accessible to 97% of the population of Bangladesh, out of which only 40% have a proper sanitation system. More than half of the population has to use unsafe drinking water, which leads to transmission of water-borne diseases like typhoid. The availability of water fluctuates highly throughout different seasons, with monsoons bringing the floods and the cooler season bringing the droughts. The Bengalis have very little control over their water supply because the great rivers, named Brahamputra, Meghna, and Ganges, originate in different countries [18].

The transmission of typhoid occurs through faecal-oral route, with hygienic measures largely involved in its control and prevention [19]. According to a study in 2020, there is a strong relationship between handwashing before eating and eating street foods from vendors with incidence of typhoid fever [20]. Street foods are ready-to-eat foods sold by vendors in public places [21].According to WHO, food handling personnel play a massive role in ensuring food safety [22].Various factors are involved in the initial contamination and subsequent transmission of different infections. One of the many factors can be the vendor location which can lead to improper food handling and improper waste disposal. Moreover, the raw materials, including water and vegetables, being used without processing are a major risk for the transmission of various pathogens including Salmonella [23].

Healthcare of rural Bangladesh is facing a massive crisis with acute shortages of doctors, nurses and technicians resulting in people turning to traditional healers. According to the annual health bulletin published by the health ministry, in 2019, it was recorded there were only 6 doctor, nurses and midwives employed in Bangladesh for every 10,000 people [24], lack of medical personnel poses a burden as the basic requirement of adequate and timely healthcare for all Bangladesh citizens is not meet [24].According to the government's Directorate General of Health Services, Upazilla Health Complexes (UHCs) are the first referral health facility at primary level of care in Bangladesh. It is quite typical in most of the UHCs where there are many vacancies, no basic facilities and maintenance, no emergency backup of electricity [25].

Salmonella Typhi has an ever-changing resistance to antibiotics. Back in 1940s when antibiotics for typhoid were first introduced, the mortality rate dropped from 26% to 1% only. In the next 20 years, the cases resistant to first line drugs, called multidrug-resistant (MDR) strains, were reported. In 1990s an aggressive strain, H58, was reported which not only evolved genetically for antibiotic resistance but also spread rapidly. The recent strain to be reported is extensively drug-resistant (XDR) strain which is resistant to both first- and second-line drugs and susceptible to only few which are difficult to access in countries like Bangladesh [26].These evolved strains add to the economic burden and the cost of management and treatment of illnesses. According to a study in 2020, the median cost of illness per case of typhoid from the patient and caregiver perspective was US$64.03. From the healthcare provider perspective, the average cost was US$58.64. Median direct and medical and nonmedical costs per case were 3% of annual labour income [27].

## 3 Efforts and recommendations

Poor sanitation infrastructure, high open defecation numbers, contaminated water sources lead to the rampant spread of typhoid infections amongst the masses. In 2012 to tackle this problem the government of Bangladesh successfully persuaded The World bank to commit $ 43 million in funds for the Bangladesh Rural Water supply and Sanitation Project. Its aim was to increase the safe water supply and hygienic conditions in the rural areas of Bangladesh. This project aimed to provide 924,000 people with access to improved water resources. 14000 community water points were constructed and rehabilitated. Under this project 28000 new piped household water connections were established and 275000 people were aimed to be provided with access to hygienic latrines. World Bank also committed $ 144 million to The Chittagong Water Supply Improvement and Sanitation Project. The project was aimed to support the establishment of a long term sustainable safe drinking water supply, sanitation, drainage infrastructure in Chittagong, one of the densely populated urban centre of Bangladesh. The project intends to improve safe water supply to a total of 150,000 people. The construction of a water treatment plant, transmission and distribution systems continue under the Chittagong Water Supply Improvement Project. One of the goals of the project is to provide technical assistance needed to develop sanitation and drainage master plans in Chittagong [28].

The coalition against typhoid, a non-governmental organisation, based at Sabin Vaccine Institute working to prevent typhoid and other invasive salmonellosis through research, advocacy, and education. They are working together for the introduction of Typhoid Conjugated Vaccine (TCV's) and improved water, sanitation and hygiene interventions in Bangladesh to reduce the burden and impact of typhoid [29].

Typhoid is often misdiagnosed as malaria or dengue to similar clinical symptoms. An important step in diagnosing this disease is improving the diagnostic techniques. At one of their research laboratories in Dhaka the Icddr, b's team are working to develop a diagnostic kit, TypoKit, which will not only be rapid but more sensitive and specific. It will be used in low-resource setting which can be beneficial in the isolated rural populations of the country [30]. With the financial support of Merieux foundation The Rodolphe Merieux Laboratory of Chittagong was opened in 2015. It is currently focusing on typhoid fever research and excels in lab diagnostics [31].

WASH Interventions i.e., Safe water, sanitation and hygiene are critical for preventing the spread of typhoid. A study from a cluster randomized study in Mirpur, Dhaka has concluded that participant who had better WASH households i.e., houses with at least one private toilet, water filter and an access to a safe drinking water source, had a 37% reduction in risk of contracting blood culture-confirmed typhoid, regardless of Typhoid Conjugated Vaccine status. According to the study a combination of both Better Wash and TCV had the lowest risk (a 37% less risk) of contracting typhoid infection. These findings suggest simple and inexpensive improvements like installation of private toilets, water filters, education about basic hygiene practices such as regular hand washing integrated with TCV's have a huge potential to reduce the risk of typhoid infections. Since Typhoid is most common in children under the age of 15 the government should start to work alongside primary schools and Madrassa to educate the children about the safe basic hygienic practices. The concerned authorities should also take into consideration about promoting campaigns educating masses about importance of vaccination through TV commercials, social media sponsored posts and distribution of Flyers and Posters in Public places. It is important that the public be informed about the mode of spread of typhoid fever, through all available channels. Messages given through these campaigns must be carefully prepared taking the local culture, traditions, and beliefs into considerations. The government should also ensure the proper chlorination of water specially in the typhoid endemic areas. There should be more provision of facilities for disposal of human excreta. In areas where such facilities still haven't been provided, digging of pit or bore hole latrines constitute a temporary practical solution. The government should also encourage households to take their own steps to disinfect drinking water.

In high-risk typhoid endemic nations like Bangladesh the role of vaccines alongside the wash interventions are critical. According to a study conducted in Mirpur, Dhaka the new TCV developed in 2017 prevents 85% of the cases among the children aged between 9 months and 15 years. This conjugate vaccine requires only one dose and is more effective and longer lasting than other typhoid vaccines. TCV will be a gamechanger for not only preventing morbidity and mortality but also to limit the spread and evolution of XDR typhoid. The Ministry of Health and Family Welfare should Introduce TCV into routine immunization programmes for the children of Bangladesh. The Vaccination against typhoid should be made mandatory for everyone. This can be achieved by carrying out free of cost vaccination drives in school, colleges, universities, offices, shopping malls and community centres etc. TCVs will prevent 66.7 million cases and 826,000 deaths due to typhoid. More than 80% of drug-resistant typhoid cases and deaths would be prevented [32].

Based on the analysis of typhoid isolates collected by The Surveillance for Enteric Fever in Asia Project, there has been an alarming increase in the level of antibiotic resistance against typhoid. With this growing threat of antibiotic resistant strains typhoid is becoming increasingly difficult to treat. There is an urgent public health need to expand preventive measures that can slow the development of drug resistance. First, strict laws should be implemented to reduce unnecessary antibiotic prescription. Azithromycin is one of the only few drugs which is still effective against XDR typhoid. Doctors should limit the prescription of Azithromycin only to the critically ill patients. Most of the people stop their medication course once the symptoms of the disease start fading away without understanding the threat drug resistance poses to the public health worldwide. To avoid this physician should educate their patients on the serious consequences of antibiotic resistance and importance of completing the prescribed course of antibiotics. It is a common practice for people in rural and less educated urban settings to get medications from local pharmacies without a verified prescription. Government should introduce strict laws which prohibit the pharmacy owners to provide medications such as Azithromycin without a verified prescription.

With the increase in the drug resistance strains of typhoid more expensive and potent drug are required for treatment which has resulted in an increase the cost for treatment. Along with this due to poor rural healthcare system patients must travel long distances to the urban cities which is an added cost to their already exhausted economic conditions. A relief can be provided to the patients suffering from this enteric disease if the government invests in new diagnostic laboratories. Such Laboratories which can do blood culture confirmation can save a lot of money that would otherwise been spent on the misdiagnosed disease [27]. Apart from this the government should also invest on telemedicine. Doctors can provide online consultation and virtual medical assistance to the patients. This can make healthcare accessible to more people in rural areas and cut down transportation costs. Lastly more doctors should be encouraged to work in rural areas of the country especially those which are the worst hit by the typhoid outbreak. This can be done by promising them better salaries, working conditions, recreational facilities etc.

The government should also start a programme of careful surveillance countrywide to ensure that sporadic cases of typhoid fever are promptly detected. An intensive search can help to identify and treat chronic carriers of the disease and save communities from the outbreak of this disease.

The best approach to reducing the burden of typhoid and other waterborne disease is to ensure the supply of uncontaminated safe drinking water to all households. This requires not only the construction of new water and sewage treatment plants but also the maintenance of the existing ones. Most of the plants in the country are either understaffed or the staff working at such plants are poorly trained. One of the major reasons for the poor functionality of the existing plants lack of political will, lack of finance and institutional systems which fail to provide the knowledge, skills and systems required for effective operation and management. The government should find substantial funding which can be used to pay the salaries of new administrative and technical personal to run these understaffed water treatment plants efficiently. There should also be a focus on setting new vocational training centres where the staff can get the technical expertise required to run these facilities appropriately [33].

According to multiple studies street food served in Bangladesh is contaminated with bacteria which can cause diseases like typhoid, cholera and shigella. Fresh juices, a popular street food option, were sampled for its bacterial load, *Staphylococcus, Shigella, Pseudomonas and Vibrio* were detected amongst many others [34]. After conducting antibiotic sensitivity testing on each bacterium, some bacteria were found to be multi-drug resistance which poses a great threat to the locals due to limited drug treatment options [34]. The contamination is due to the use of dirty water and poor hygiene practices. Many people in developing nations like Bangladesh rely on street food as it is more affordable and convenient. To tackle the problem of typhoid spread through unhygienic street food, the government should make sure that the street food vendors should only be allowed to operate after they have passed a physical fitness test and completed a basic hygiene training. The street cart owners should be given strict instructions on the necessary protocols to prepare food such as using sanitizers frequently, not mixing cooked food with raw meat, preparing food in properly covered area instead of closed area etc. Such practices can minimize the risk of bacterial infections. There should be frequent inspections by the relevant health and food authorities to inspect if the street vendors are following the safe hygienic practices to prepare food and those who fail to comply with the basic hygiene practices at their stalls should be shut down until they maintain the health and safety standards.

## Ethical approval

N/A.

## Sources of funding

None.

## Author contribution

Yumna Salman conceived the idea, wrote the introduction, and organised references; Narmeen Hashmi wrote the challenges and implications; Hanzla Asim wrote the efforts and recommendation; Zarmina Islam edited the revised draft; Mohammad Yasir Essar and Ariful Haque made the critical comments and revision. All authors revised and approved the final draft.

## Consent

N/A.

## Registration of research studies

1. Name of the registry: N/A.

2. Unique Identifying number or registration ID: N/A.

3. Hyperlink to your specific registration (must be publicly accessible and will be checked): N/A.

## Guarantor

Md Ariful Haque.

## Provenance and peer review

Not commissioned, externally peer reviewed.

## Declaration of competing interest

None declared.

## Abbreviations

Coronavirus disease 2019: COVID-19
*Salmonella typhi*: S.typhi
Upazilla health complexes: UHC
Multi-drug resistance: MDR
Extensively drug-resistant: XDR
Typhoid conjugated vaccine: TCV
